# An indeterminate mucin-producing cystic neoplasm containing an undifferentiated carcinoma with osteoclast-like giant cells: a case report of a rare association of pancreatic tumors

**DOI:** 10.1186/s12876-015-0391-2

**Published:** 2015-11-18

**Authors:** Marco Chiarelli, Angelo Guttadauro, Martino Gerosa, Alessandro Marando, Francesco Gabrielli, Matilde De Simone, Ugo Cioffi

**Affiliations:** 1 Department of Surgery, Ospedale Alessandro Manzoni, Lecco, Via dell’Eremo 9/11, 23900 Lecco, LC Italy; 2 Department of Surgery, University of Milan-Bicocca, Istituti Clinici Zucchi, Via Zucchi, 24, 20900 Monza, MB Italy; 3 Department of Pathology, Ospedale Alessandro Manzoni, Lecco, Via dell’Eremo 9/11, 23900, Lecco, LC Italy; 4 Department of Surgery, University of Milan, Milan, Italy

**Keywords:** Mucinous cystic neoplasm, Intraductal papillary mucinous neoplasm, Osteoclast-like giant cells carcinoma, Pleomorphic giant cell carcinoma, Pancreas

## Abstract

**Background:**

Only few case reports of mucinous cystic pancreatic neoplasm containing an undifferentiated carcinoma with osteoclast-like giant cells have been described in the literature. In the majority of cases this unusual association of tumors seems related to a favorable outcome. We present the second case of an indeterminate mucin-producting cystic neoplasm containing an area of carcinoma with osteoclast-like giant cells. The specific features of the two histotypes and the rapid course of the disease make our clinical case remarkable.

**Case presentation:**

A 68 year old female came to our attention for a pancreatic macrocystic mass detected with ultrasonography. Her past medical history was silent. The patient reported upper abdominal discomfort for two months; nausea, vomiting or weight loss were not reported. Physical examination revealed a palpable mass in the epigastrium; scleral icterus was absent. Cross-sectional imaging showed a complex mass of the neck and body of the pancreas, characterized by multiple large cystic spaces separated by thick septa and an area of solid tissue located in the caudal portion of the lesion. The patient underwent total pancreatectomy with splenectomy. Pathological examination revealed a mucinous cystic neoplasm with a component of an undifferentiated carcinoma with osteoclast-like giant cells. Because of the absence of ovarian-type stroma, the lesion was classified as an indeterminate mucin-producing cystic neoplasm of the pancreas. The immunohistochemical studies evidenced no reactivity of osteclast-like giant cells to epithelial markers but showed a positive reactivity to histiocytic markers. Numerous pleomorphic giant cells with an immunohistochemical sarcomatoid profile were present in the undifferentiated carcinoma with osteoclast-like giant cells. A rapid tumor progression was observed: liver metastases were detected after 4 months. The patient received adjuvant chemotherapy (Gemcitabine) but expired 10 months after surgery.

**Conclusion:**

Our case confirms that the presence of a solid area in a cystic pancreatic tumor at cross-sectional imaging should raise a suspicion of malignant transformation. The lack of ovarian-type stroma in a pancreatic mucinous cystic neoplasm and the presence of pleomorphic giant cells in an undifferentiated carcinoma with osteoclast-like giant cells could be a marker of a poor prognosis.

## Background

The World Health Organization (WHO) classifies pancreatic mucin-producing cystic tumors into two different pathological entities: mucinous cystic neoplasm (MCN) and intraductal papillary mucinous neoplasm (IPMN) [1]. MCNs are thick-walled macrocystic tumors characterized by the absence of communication with ductal system. The distinctive histological feature of an MCN is a columnar mucin-producing epithelium supported by an ovarian-type stroma; this neoplasm occurs typically in premenopausal women [1–3]. IPMNs are intraductal tumors characterized by epithelial papillary proliferation and mucin hypersecretion causing a typical cystic dilatation of the pancreatic ductal system. IPMNs occur frequently in the head of the gland and are characterized by the communication between the tumor and the pancreatic ducts [1, 3]. IPMN has an equal gender distribution and occurs frequently in the seventh decade of life [1]. On the basis of epithelial atypia and invasiveness, cystic neoplasms are classified as adenoma, non-invasive carcinoma and invasive carcinoma [1, 3].

The undifferentiated carcinoma is a rare and aggressive form of pancreatic neoplasm. The WHO classification describes two distinct histological types: osteoclast-like giant cell carcinoma (OGCC) and pleomorphic giant cell carcinoma (PGCC) [1]. PGCC shows a sarcomatoid growth pattern, characterized by the presence of pleomorphic mononucleated and multinucleated bizarre giant cells [1, 4]. OGCC is composed of spindle-shaped or ovoidal mononuclear cells and scattered giant cells with multiple small regular nuclei [1, 4].

In 1981, Posen described for the first time the association of a giant cell tumor of the osteoclastic type with a mucous secreting cystic pancreatic neoplasm and since then only 12 cases have been reported [5, 6]. In a recent review Wada et al. analyzing all the cases in the literature, reported a favourable prognosis for this particular tumor association [6]. We present the second case in the literature of a patient with an indeterminate mucin-producting cystic neoplasm containing a single area of undifferentiated carcinoma with osteoclast- like giant cells, characterized by a rapid disease progression.

## Case presentation

### Clincal history and treatment

A 68 year old caucasian female with a pancreatic cystic neoplasm diagnosed on ultrasonography (US) presented at our Institute in December 2013. Her past medical history was silent. No abuse of ethanol or smoking was reported. The patient reported upper abdominal discomfort for 2 months; nausea, vomiting or weight loss were not referred. The physical examination revealed a palpable mass in the epigastrium; scleral icterus was absent. No other clinical abnormalities were detected.

The US showed a 5 × 6 cm pancreatic macrocystic lesion localized in the body of the gland. Laboratory tests showed that blood cell counts and C-reactive protein were normal. Levels of serum glucose, creatinine, albumin, aminotransferase, gamma-glutamyl transferase, alkaline phosphatase and lipase were all within normal limits. The serum levels of the following tumor markers were elevated: carcino-embryonic antigen (CEA) was 196 ng/ml (normal range <5 ng/ml) and carbohydrate antigen 19-9 (CA 19-9) was 66 U/ml (normal range <33 U/ml).

The patient underwent a triphasic multidetector computed tomography (CT) scan and a T1, T2-weighted and DWI contrast enhanced magnetic resonance imaging (MRI) with intravenous Gadolinium administration.

**Fig. 1:**
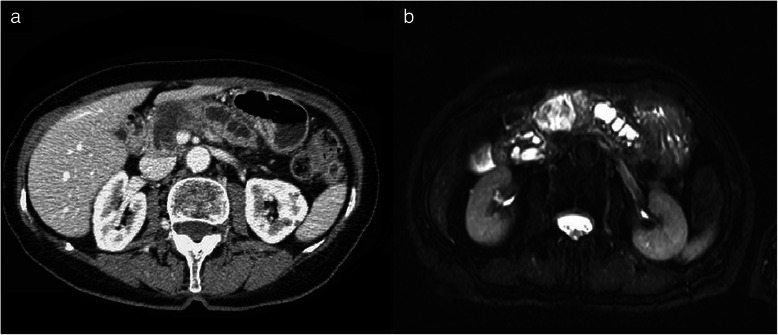
**a** Contrast enhanced CT scan demonstrates a complex pancreatic macrocystic mass involving the neck and the body of the pancreas with a peripheral solid portion. **b** Axial T2-weighted MRI shows the high-intensity central cystic portion of the mass with inner irregular septa and the peripheral less intense solid tissue determining main duct dilatation

The CT scan showed a large complex cystic and solid mass measuring 5 × 3 × 6 cm involving the neck and the body of the pancreas (Fig. 1a). The tumor had a predominant central component characterized by multiple large cystic spaces (up to 3 cm in diameter) separated by thick septa. A 2 cm area of low attenuation solid tissue was located between cyst walls in the caudal portion of the lesion. The main pancreatic duct was enlarged in its entirity (diameter 12 mm) with a more marked dilatation in the body and tail region. Irregular calcifications were evident in the peripheral portion of the tumor. There was compression of superior mesenteric vein by the mass with a minimal infiltration of the splenic vein. The CT scan of the chest and abdomen did not show any lymphadenopathy, hepatic and pulmonary metastasis.

MRI confirmed the complex pancreatic lesion featured by a large cystic portion with inner irregular septa and a peripheral solid area measuring 2 cm in the lower portion of the tumor (Fig. 1b). Cholangiopancreatographic images did not demonstrate a communication between the enlarged main pancreatic duct and the cystic mass; ectatic branch ducts were present in the body and tail region.

At laparotomy a large cystic mass involving the body and the neck of pancreas was confirmed. Further exploration showed an infiltration of the head of the gland but ruled out neoplastic spreading to adjacent organs and lymph nodes; the splenic vein was infiltrated by the mass at superior mesenteric veinous junction. Patient underwent total pancreatectomy with splenectomy and lymph node dissection. A US performed on 7th post-operative day ruled out any intra-abdominal fluid collection. Patient developed post-pancreaectomy diabetes which was difficult to control by insulin therapy. The patient was discharged 14 days after the operation.

### Histopathological findings

Macroscopically the pancreas was involved by a 6 cm well-circumscribed round mass localized in the neck and the body with infiltration of the head; stomach, duodenum, common bile duct and spleen were not infiltrated. Externally the tumor presented a smooth reddish surface and the pancreatic parenchyma not involved by the lesion appeared fibrotic. On cut-section the lesion showed multilocular macrocystic spaces separated by fibrous septa and filled by a clear viscous mucoid fluid. The main pancreatic duct was ectatic but it was not possible to recognize a communication with the cystic lesion. A 2 cm single yellow solid area was located in the caudal portion of the mass.

**Fig. 2:**
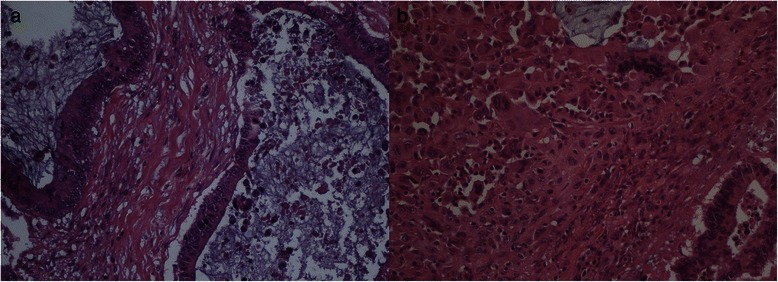
**a** Microscopically the tumor is characterized by a predominant cystic lesion composed of a columnar mucinous epithelium with atypical nuclei, numerous mitoses and stromal invasion. An ovarian-type stroma is absent (hematoxylin/eosin staining, 20x). **b** The solid area contains mononuclear spindle-shaped cells, pleomorphic giant cells and scattered multinucleated osteoclast-like giant cells (hematoxylin/eosin staining, 20x)

Microscopically the tumor was principally composed of a cystic mucinous part characterized by a columnar mucinous epithelium with atypical nuclei, copious mitoses and stromal invasion. It was not possible to find an ovarian-like stroma (Fig. 2a). In the caudal part of the neoplasm there was a solid area containing mononuclear spindle cells, associated with pleomorphic giant cells (PGCs) and scattered multinucleated osteoclast-like giant cells (OGCs) (Fig. 2b).

The surgical margins were negative for neoplastic infiltration. No lymph node metastases were shown. There was no evidence of perineural or vascular infiltration.

**Fig. 3:**
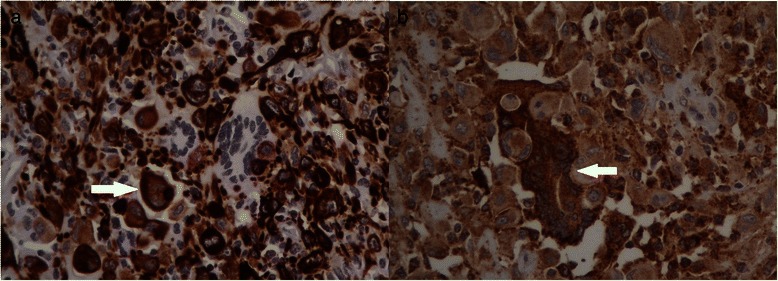
**a** Mononuclear spindle-shaped cells and pleomorphic giant cells (*arrow*) are immunoreactive for epithelial markers (CK8/18), while multinucleated osteoclast-like giant cells are negative (40x). **b** Mononuclear spindle-shaped cells and pleomorphic giant cells stain weakly for histiocytic marker CD68; in contrast, multinucleated osteoclast-like giant cells show an intense immunoreactivity for CD68 (*arrow*) (40x)

The mononuclear spindle cells and pleomorphic giant cells were immunoreactive for epithelial markers including CK8/18 and CK19, and for vimentin and expressed a weak immunoreactivity for CD68 and a focal immunoreactivity for actin and CD10. The proliferative index (Ki-67) of the mononuclear spindle cells and pleomorphic giant cells was high (approximately 30 %) (Fig. 3a). Multinucleated osteoclast-like giant cells intensely expressed histiocytic marker (CD68) and vimentin but they were negative for epithelial markers (CK8/18 and CK19) and actin. In these cells Ki-67 immunoreaction was negative (Fig. 3b).

The histopathological findings supported the diagnosis of indeterminate mucin-producing cystic neoplasm with a component of osteoclast-like giant cell carcinoma.

### Follow-up

Two months after surgery serum CEA and CA 19-9 decreased to 83 ng/ml and 31 U/ml, respectively. Nevertheless after 4 months serum CEA increased to 144 ng/ml and hepatic metastases were founded at follow-up CT scan. Gemcitabine single agent chemotherapy was started (1,00 mg/m^2^ weekly × 7, then weekly × 3 every 4 weeks). CT scan performed 8 months after surgery showed the progression of liver metastases and appearance of lung metastases. The patient expired 10 months after surgery.

## Discussion

International Consensus Guidelines have established how to diagnose and manage pancreatic cystic tumors [7]. Although their clinical, radiological and pathological features have been defined, in some cases the differential diagnosis between mucinous forms is difficult [8].

In our case the assessment of a correct preoperative diagnosis was difficult due to the clinical and radiological findings. Patient’s age and main duct dilatation were elements in favor of IPMN, while female gender and the involvement of the body of the gland leaned towards MCN. However the diagnosis of IPMN was excluded as there was no communication between the cystic mass and the main pancreatic duct.

Tumor size, peripheral calcifications, irregular septa, and the presence of an intramural nodule pointed towards malignant transformation and led to surgical resection. Indeed all these radiological findings are very frequently associated with malignant histology [9].

On the basis of microscopic criteria (nuclear atypia, number of mitoses, and invasion into the stroma), the lesion should be categorized as a mucinous cystoadenocarcinoma. Nevertheless the absence of ovarian-type stroma made us to classify it as an indeterminate mucin-producing cystic neoplasm of the pancreas [7]. In the literature the lack of ovarian-type stroma is more frequent in postmenopausal women and MCNs that do no express ovarian-type stroma have a worse prognosis compared to those which have ovarian-type stroma [2, 10].

OGCC is rare pancreatic neoplasm: a US population study based on Surveillance, Epidemiology and End Result (SERR) database evidenced that its incidence is 11 % of all undifferentiated carcinomas of the pancreas [11].

In the past decades the histogenesis of OGCC was debated, but the more recent immunohistochemical studies demonstrate that OGCs present no reactivity to epithelial markers and show a positive reactivity to histiocytic marker CD68; by contrast, spindle-shaped mononuclear cells express epithelial markers [12, 13]. The immunoreactivity for Ki-67 reveals a low proliferative activity of OGCs and a high proliferative activity for mononuclear cells [13]. These data, as our findings, confirm the hypothesis that spindle-shaped mononuclear cells are neoplastic elements, while OGCs are not neoplastic and may have a histiocytic lineage [12, 13].

In our case numerous PGCs were detected in the solid area of the neoplasm. These cells are frequently characterized by immunoreactivity for epithelial markers like cytokeratins and CEA and by a high proliferative index; in some cases, PGCs express mesenchymal markers like vimentin [14]. These data support the hypothesis that PGCs are undifferentiated neoplastic cells derived from epithelial elements with a sarcomatoid profile. These histological changes are typical of the epithelial-mesenchimal transition which is a marker of tumor de-differentiation and invasiveness [15].

The simultaneous presence of PGCs and OGCs within the same tumor indicates a possible overlap between the two histological types: some authors propose to classify the mixed giant cell carcinoma as a different histopathological entity containing both osteoclastic and pleomorphic giant cells in significant proportion [4, 14, 16].

Incongruous data is there in the literature about OGCC prognosis. Early reports based on single case suggested that it might have a better outcome than ordinary ductal carcinoma [17]. However in a series of nine cases all patients but one died within 1 year from diagnosis [12]. In a retrospective analysis of 15 patients with anaplastic carcinoma of the pancreas, all long-term survivors presented a neoplasm containing OGCs and the median survival was significantly better in this histological type [18]. In two small series, OGCCs with a high proportion of PGCs were associated with a shorter survival [13, 19] and in an overall analysis of the few reported cases, the presence of a cell population expressing epithelial markers seemed to predict a worse prognosis [20].

Ours is the second case described in the english literature of an indeterminate mucin-producting cystic neoplasm containing an area of undifferentiated carcinoma with osteoclast-like giant cells. In a review that analyzed all cases of MCN associated with OGCC, 10 of 12 patients were alive at follow-up [6]. By contrast our report was remarkable for the rapid progression of the tumor. It is interesting to note that both cystic and solid components of the neoplasm presented histological features of malignancy. Only further studies based on large series with longer follow-up will clarify if the absence of ovarian-type stroma and the presence of PGCs could be related with the outcome of these tumors.

## Conclusions

There are only few case reports in literature of an MCN with a component of OGCC. Due to limited data it is difficult to establish accurately radiological presentation and clinical outcome of these patients. Our case confirms that the presence of solid area in a cystic tumor at cross-sectional imaging should raise a suspicion of malignant transformation. In conclusion, the peculiar histological features of the two neoplasms and the rapid course of the disease make our case remarkable.

### Consent

Written informed consent was obtained from the patient for publication of this Case report and any accompanying images. A copy of the written consent is available for review by the Editor of this journal.

## Abbreviations

WHO: World Health Organization
SEER: Surveillance, Epidemiology and End Result
MCN: Mucinous cystic neoplasm
IPMN: Intraductal papillary mucinous neoplasm
OGCC: Osteoclast-like giant cell carcinoma
PGCC: Pleomorphic giant cell carcinoma
OGCs: Osteoclast-like giant cells
PGCs: Pleomorphic giant cells
CEA: Carcino-embryonic antigen
CA 19-9: Carbohydrate antigen 19-9
US: Ultrasonography
CT: Computed tomography
MRI: Magnetic resonance imaging
CK8/18: Cytokeratin 8/18
CK19: Cytokeratin 19
CD68: Cluster of differentiation 68
CD10: Cluster of differentiation 10
